# DeepHistone: a deep learning approach to predicting histone modifications

**DOI:** 10.1186/s12864-019-5489-4

**Published:** 2019-04-04

**Authors:** Qijin Yin, Mengmeng Wu, Qiao Liu, Hairong Lv, Rui Jiang

**Affiliations:** MOE Key Laboratory of Bioinformatics; Bioinformatics Division, Beijing National Laboratory for Information Science and Technology, Tsinghua University, Beijing, 100084 China

**Keywords:** Histone modification, Chromatin accessibility, Deep learning, Sequence analysis, Genetic variation

## Abstract

**Motivation:**

Quantitative detection of histone modifications has emerged in the recent years as a major means for understanding such biological processes as chromosome packaging, transcriptional activation, and DNA damage. However, high-throughput experimental techniques such as ChIP-seq are usually expensive and time-consuming, prohibiting the establishment of a histone modification landscape for hundreds of cell types across dozens of histone markers. These disadvantages have been appealing for computational methods to complement experimental approaches towards large-scale analysis of histone modifications.

**Results:**

We proposed a deep learning framework to integrate sequence information and chromatin accessibility data for the accurate prediction of modification sites specific to different histone markers. Our method, named DeepHistone, outperformed several baseline methods in a series of comprehensive validation experiments, not only within an epigenome but also across epigenomes. Besides, sequence signatures automatically extracted by our method was consistent with known transcription factor binding sites, thereby giving insights into regulatory signatures of histone modifications. As an application, our method was shown to be able to distinguish functional single nucleotide polymorphisms from their nearby genetic variants, thereby having the potential to be used for exploring functional implications of putative disease-associated genetic variants.

**Conclusions:**

DeepHistone demonstrated the possibility of using a deep learning framework to integrate DNA sequence and experimental data for predicting epigenomic signals. With the state-of-the-art performance, DeepHistone was expected to shed light on a variety of epigenomic studies. DeepHistone is freely available in https://github.com/QijinYin/DeepHistone.

## Background

Histone modifications, as covalent post-translational modifications (PTMs) to histone proteins, have been recognized as one of the major driving forces alters chromatin structures since the early 1960s [1]. Enabled by such innovative techniques as X-ray crystallography, it has been gradually clear that the modification of histone amino (N)-terminal tails would affect inter-nucleosomal interactions, alter the overall chromatin structure or recruit histone modifiers, and eventually impact gene expression [2]. It has also been known that histone modifications, including methylation, acetylation, phosphorylation, ubiquitylation and sumoylation, act in a variety of biological processes such as chromosome packaging [3, 4], transcriptional activation and inactivation [5–7], as well as DNA damage and repair [8]. Therefore, quantitative detection of histone modifications would provide useful information for not only a better understanding towards epigenetic regulation of cellular processes but also the development of drugs targeting on histone modifying enzymes [9].

Histone modifications are mainly profiled by such high-throughput experimental techniques as chromatin immunoprecipitation followed by sequencing (ChIP-seq) [10]. For example, Barski et al. generated high-resolution maps for the genome-wide distribution of 20 histone lysine and arginine methylations and identified typical patterns of histone methylations exhibited at promoters, insulators, enhancers, and transcribed regions [11]. Whole-genome profiling of DNA regulatory elements, their relationship to target genes, their properties of histone modifications, and their features of chromatin accessibility, were conducted by the Encyclopaedia of DNA Elements (ENCODE) project [12]. Even larger scale global maps of regulatory elements in 111 reference human epigenomes, together with chromatin accessibility and gene expression information, were established by the Roadmap Epigenomics Consortium [13]. These abundant resources provided new insights into the function of histone modification and chromatin organization in genome, demonstrated the central role of epigenomic information for understanding gene regulation and cellular differentiation, and opened a door towards deciphering mechanisms of human disease.

Nevertheless, it is still too expensive and time-consuming to establish a landscape of histone modifications purely relying on biological experiments, due to the large number of cell types and known histone markers. It is, therefore, reasonable to take advantage of computational methods to predict histone modifications, complementing experimental approaches and facilitating the understanding of DNA signatures and modifications that contribute to gene expression. Towards this objective, Benveniste et al. designed a logistic regression model to predict histone modifications from transcription factor-binding profiles and recapitulated the importance of interactions between transcription factors and chromatin-modifying enzymes to gene expression [14]. Karlic et al. elucidated the correlation between histone modification levels and gene expression and designed a linear regression model to predict gene expression relying on a small number of histone modifications [15].

In the recent years, deep learning has been successfully incorporated into a variety of bioinformatics studies. For example, Alipanahi et al. proposed a convolutional neural network (CNN) named DeepBind to predict binding proteins and showed higher prediction power than traditional classifiers [16]. Zhou and Troyanskaya designed a model called DeepSEA to learn DNA regulatory signatures via a CNN from epigenomic data [17]. Quang and Xue combined a CNN and a bi-directional long short-term memory network to predict functions of DNA sequences and named their method DanQ [18]. Min et al. proposed a deep CNN model called DeepEnhancer to predict enhancers purely from DNA sequences [19]. Liu et al. designed a hybrid neural network to predict chromatin accessibility from sequence [20]. Min et al. further developed a representation learning formulation to embed *k*-mers into a low dimension space and then used the resulting vectors to predict chromatin accessibility via a deep neural network [21]. The success of these methods suggests that deep learning is a powerful technique in genomic studies. However, all these methods rely purely on DNA sequence information, which apparently lacks the power of making predictions in a cell line-specific manner, because DNA sequences are identical in different cell lines. To overcome this limitation, hybrid deep learning methods have been proposed and shown visible improvement in specific research by combining sequence information and biological experimental data. For instance, a recently proposed method named DeepTACT combined DNA sequences and chromatin accessibility to predict high-resolution chromatin contacts from promoter capture Hi-C data and achieved state-of-the-art performance [22].

Motivated by the above understanding, we purposed a deep learning approach named DeepHistone to predict histone modification by integrating DNA sequence information and chromatin accessibility data. The rationale for our method is to capture regulatory signatures from DNA sequences, while taking advantage of the compact relationship between histone modifications and chromatin accessibility to further improve the prediction performance. Through a serial of comprehensive validation experiments, we demonstrated that DeepHistone is superior to several baseline methods in the prediction of modification sites specific to different histone markers, not only within an epigenome but also across epigenomes. Besides, we illustrated that sequence signatures automatically extracted by our deep learning model was consistent with known transcription factor binding sites. As a potential application, we finally showed the possibility of our method in distinguishing functional single nucleotide polymorphisms (SNPs) from their nearby genetic variants.

## Materials and methods

### Data sources

We downloaded peak files of 7 histone modification markers for 21 human epigenomes from the Roadmap Epigenomics Project [13]. As shown in Table 1, the 7 markers, including H3K4me3, H3K4me1, H3K36me3, H3K27me3, H3K9me3, H3K27ac, and H3K9ac, are regarded as the most important markers that have been verified to be associated with such specific functional regions as enhancers and promoters in the genome [23]. The criterion for selecting an epigenome is that ChIP-seq assays should be performed for all the 7 markers for the tissue or cell line corresponding to the epigenome.

**Table 1:** The 15 epigenomes used in this research

Epigenome ID (EID)	Anatomy	Histone marker							
		H3K4me1	H3K4me3	H3K27me3	H3K36me3	H3K9me3	H3K9ac	H3K27ac	Total
E003	ESC	342,503	213,546	231,823	424,644	322,656	155,558	236,273	1,460,537
E005	ESC_DERIVED	433,887	166,189	107,589	456,459	287,790	71,753	258,471	1,349,880
E006	ESC_DERIVED	462,960	163,290	130,314	487,545	278,273	168,264	289,090	1,547,640
E007	ESC_DERIVED	263,177	159,076	69,494	192,670	149,941	294,433	118,545	957,424
E008	ESC	147,743	208,835	80,768	336,585	234,898	168,324	129,238	1,050,986
E017	LUNG	548,594	191,906	506,666	682,735	531,686	273,512	382,596	2,302,996
E114	LUNG	651,428	362,931	483,416	585,379	216,237	381,391	430,496	1,979,536
E117	CERVIX	593,890	285,181	406,167	347,919	95,082	260,910	347,656	1,533,734
E118	LIVER	541,823	271,863	205,199	340,891	246,440	220,457	278,768	1,451,201
E119	BREAST	563,758	192,740	260,776	242,863	376,845	232,169	366,810	1,566,171
E121	MUSCLE	591,321	244,747	708,930	483,319	156,381	356,341	486,568	2,110,799
E122	VASCULAR	582,593	211,344	404,019	317,600	252,718	260,503	360,372	1,617,712
E124	BLOOD	839,651	431,883	597,823	1,098,084	570,919	268,232	655,859	2,981,094
E125	BRAIN	627,409	258,248	321,184	400,306	178,567	359,857	424,867	1,629,166
E127	SKIN	708,918	289,273	466,659	513,074	186,065	302,827	416,314	1,900,958

7,626,807 modification sites specific to the 7 histone markers were identified from the 15 epigenomes

Given a marker, an epigenome, and the peak file of the corresponding ChIP-seq experiment, we used a window of 200 bp to scan the whole human genome (hg19) with step 200 bp and regarded a window that had at least 100 bp overlap with a peak as a histone modification site. Applying this procedure to every marker and every epigenome and discarding epigenomes (total 6 epigenomes) that had only a small number of modification sites (< 50,000) for some histone markers, we identified a total of 7,626,807 sites in the human genome from 15 epigenomes, as detailed in Table 1.

For an epigenome, we further downloaded corresponding DNase-seq peak files from Roadmap. For a genomic position in a peak, we assigned the fold enrichment score of the peak, calculated by the standard pipeline of Roadmap [13], to the position, as its openness score to quantify the status of chromatin accessibility. For other genomic positions, we regarded their openness scores as zeros. By doing this for every epigenome, we obtained an openness score that was specific to the epigenome for every genomic position.

### Design of DeepHistone

**Fig. 1:**
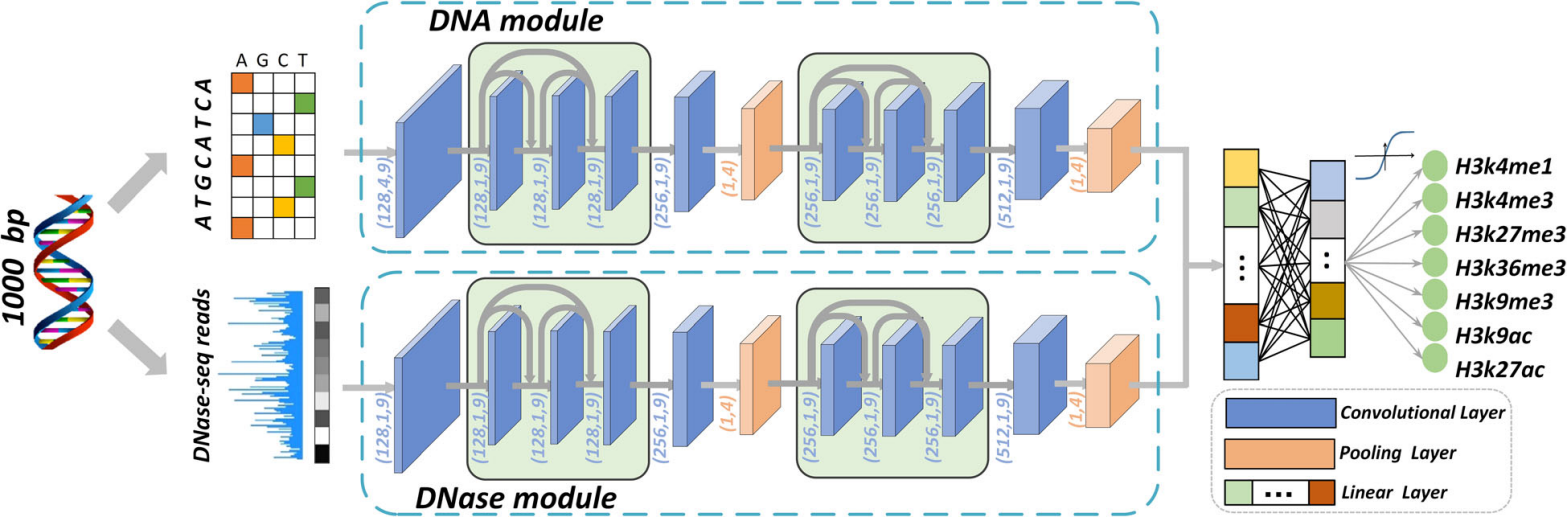
Diagram of DeepHistone. The deep neural network model consists of three modules: a DNA module, a DNase module, and a joint module. The DNA module extracts sequence information via a densely connected convolutional neural network. The DNase module deals with chromatin accessibility information using the similar architecture. The joint module combines both sequence and chromatin accessibility features to distinguish histone modification sites of a marker from those of other markers. In figure, (128,4,9) indicates there are 128 kernels, each of width 4 and length 9 in a convolutional layer and (1,4) indicates kernel width and length are set to 1 and 4 respectively in a pooling layer. Others have similar meaning

We designed a deep neural network model, named DeepHistone, to predict whether a DNA fragment in an epigenome is a site for the 7 histone markers. To achieve this aim, we first extended the input fragment upstream and downstream to obtain a region of 1000 bp centred at the fragment and then fed the resulting region to our model, which consists of three modules: a DNA module, a DNase module, and a Joint module, as illustrated in Fig. 1.

The DNA module, designed as a customized densely connected convolutional neural network [24], extracts sequence information for the input region. For this purpose, a one-hot encoding strategy is used to convert the sequence of the input region into a binary matrix. An initial convolutional layer is then adopted to scan the matrix for sequence patterns, i.e., motifs. The resulting patterns are further fed to two densely connected convolutional blocks connected in a tandem way by a convolutional layer and a pooling layer for extracting high-level features. These features, after passing through a convolutional layer and a pooling layer, are eventually fed to the joint module for the classification task. A densely connected convolutional block consists of three convolutional layers. Mediated by a batch normalization operation and a ReLU activation function, the first two layers connect to not only the subsequent layer but also all latter layers. The densely connected architecture is adopted here because recent advances in deep learning have shown that such an architecture can effectively overcome the vanishing gradient problem, strengthen feature propagation, utilize parameters more efficiently, and avoid the overfitting problem [24]. These shortcomings are common in a classical convolutional neural network, especially on tasks with small dataset. Detailed parameter settings of the DNA module are shown in Fig. 1.

The DNase module extracts chromatin accessibility information for an input region. This module has the identical architecture as the DNA module, except that an initial one-dimensional convolutional layer is used to deal with openness scores of positions in the region at the beginning.

The joint module integrates features extracted by the DNA and DNase modules to produce classification results. To achieve this objective, features extracted by these two modules are concatenated and fed to a feedforward neural network, which uses 7 sigmoid functions to predict in parallel probabilities that a region is a site for the 7 histone modification markers. Note that multiple sigmoid functions instead of a softmax function are adopted because in reality the events that a site belongs to the markers are not mutually exclusive. In other words, a site can belong to multiple markers simultaneously.

We implemented DeepHistone in Python using Pytorch [25]. The high-performance NVIDIA GeForce GTX 1080Ti GPU was used to accelerate the computation. The cross entropy loss was used as the optimal function in model training, measuring the similarity between a true distribution *p* and the prediction probability *q*, as:

$$ H\left(p,q\right)={\mathrm{E}}_p\left[\frac{1}{\log {q}_x}\right] $$

Adam [26] was used to accelerate backpropagation with default parameters, except that the initial learning rate is set to 0.001. An early stopping strategy was used to reduce the training time.

### Baseline methods

We compared the performance of DeepHistone with three baseline methods, including DeepSEA [17], DanQ [18], and gkm-SVM [27], with parameters proposed by the respective authors. Briefly, DeepSEA used three convolutional layers, a fully connected layer, and a sigmoid output layer to distinguish epigenomic sites. DanQ used a convolutional layer, a bi-directional long short-term memory layer, a fully connected layer, and a sigmoid output layer to classify DNA sequences. Gkm-SVM represented a DNA sequence as a gapped *k*-mer vector and then resorted to the widely used support vector machine (SVM) to do binary classification. We also proposed two variations of our model, named “DeepHistone (DNA-only)” and “DeepHistone (DNase-only)”. The former discards the DNase module and predicts histone modification markers using only DNA sequence information, and the later discards the DNA module and makes predictions using only chromatin accessibility data.

### Validation method and evaluation criteria

We adopted 5-fold cross-validation experiments to validate the performance of a method in predicting histone modification sites. Briefly, from ChIP-seq peak files regarding the 15 epigenomes and 7 histone markers, we identified a total of 7,626,807 modification sites. Given one of the 15 epigenomes, we partitioned all the known sites into five parts of nearly equal size. Then, in each fold of the validation, we used four parts to train a model and tested its performance on the remaining part. This procedure was repeated five times to guarantee that each site had been tested once and only once. Note that gkm-SVM is very time-consuming when compared with a deep learning method that can be accelerated by hardware (e.g., GPU). Consequently, we had to sample at random only a small number (50,000) of modification sites in the validation experiments for gkm-SVM, in order to complete the experiments in reasonable time.

Although our method can simultaneously predict whether a DNA fragment in an epigenome is a site for the 7 histone markers, a fragment has only two status for a certain marker, being a histone modification site or not. This understanding allows us to evaluate the performance of our method using the traditional formulation of binary classification. Specifically, given a histone marker, at a certain threshold of the prediction probability, we calculated the sensitivity as the fraction of its modification sites assigned a probability higher than the threshold, and the specificity as the fraction of sites not relevant to the marker and assigned a probability lower than the threshold. Varying the threshold value from 0 to 1, we were able to draw a receiver operating characteristic (ROC) curve. The area under this curve was then calculated as a criterion called auROC. Considering that the number of none-relevant modification sites for a marker is typically much larger than that of true sites, we further calculated the recall and precision at a threshold, drew a precision-recall curve by varying the threshold value, and obtained the area under this curve as another criterion called auPRC.

The rationale for our method and validation design is conceptually equivalent to using modification sites specific to a histone marker as positive set and those not relevant to the marker as negative set to train a binary classification model for the marker. However, our design has two advantages. First, instead of training 7 models for the 7 markers separately, our method can simultaneously train a model for all the 7 markers, thereby saving computational time. Second, the selection of the negative set in our design is much more stringent than such strategies as selecting DNA fragments at random from the whole genome, because modification sites for different markers may have some similar properties, e.g., GC contents, the distance to a gene, etc.

### Motif visualization

To interpret how DeepHistone captures DNA sequence patterns, we proposed the following strategy to demonstrate the relationship between known DNA binding motif and sequence patterns extracted by the first convolutional layer of DNA module. Following the literature [18, 20], we first generated a position weighting matrix (PWM) for each kernel in first convolutional layer of the DNA module by scanning along all the input sequences to find activated regions and then averaging over all the activated regions. Formally, a region **x**_*i*_ in an input sequence **s** was regarded as activated, if

$$ sum\left({\mathbf{w}}^k\cdot {\mathbf{x}}_i\right)\ge \alpha \times EAV $$

where **w**^*k*^ is the weight matrix of the *k*-th kernel, α ∈ (0, 1) a control coefficient, and *EAV* the extreme activation value of **s** defined as

$$ EAV={\max}^{\mathrm{f}\left(\right)}\left( sum\left({\mathbf{w}}^k\cdot {\mathbf{x}}_i^T\right)|\forall {\boldsymbol{x}}_i\in \boldsymbol{s}\right) $$

We set the length of a kernel to 9 and α to 0.9. We then compared extracted PWMs to the JASPAR database [28] and illustrated the results by using the tool TomTom [29] with *q*-value threshold 0.05.

### Analysis of functional implications of haQTLs

We applied DeepHistone to explore functional implications of single nucleoid polymorphisms (SNPs) related to histone acetylation quantitative trait loci (haQTLs) identified in a lymphoblastoid epigenome by the histone H3 acetylated on lysine 27 (H3K27ac) marker [30]. Given a SNP, we identified the 1000 bp DNA sequence centred at the SNP position and predicted two probabilities, *p*^*ref*^ and *p*^*alt*^, that indicate the degree that the reference and alteration sequences being a histone modification site for the H3K27ac marker, respectively. Following the literature [20], the absolute value of the different between the two predictions was then defined as the functional implication score, Δ*p* =  ∣ *p*^*alt*^ − *p*^*ref*^∣ for the SNP.

## Results

### DeepHistone accurately predicts histone modification sites

**Fig. 2:**
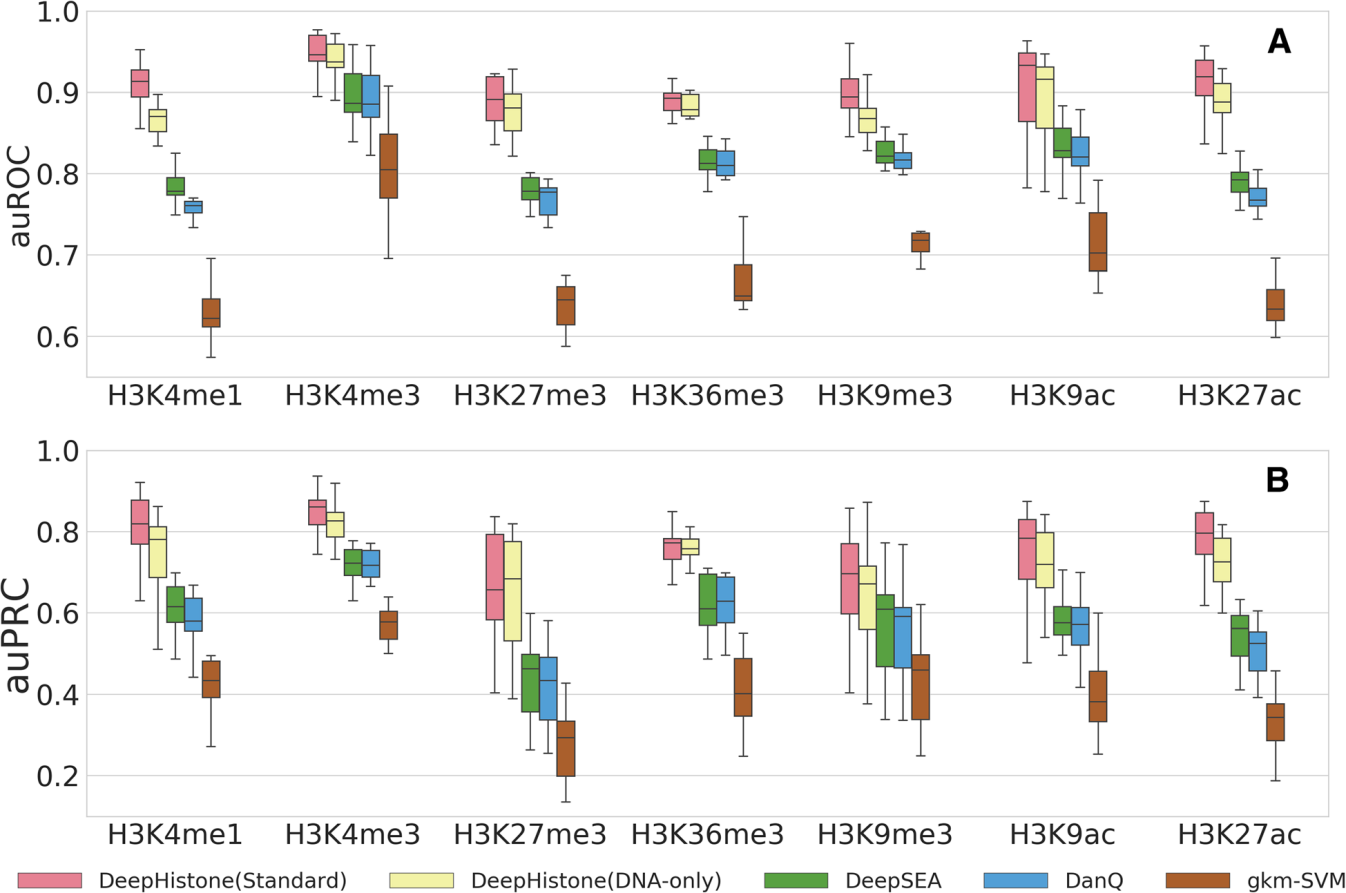
Performance of different methods in terms of auROC (**a**) and auPRC (**b**). In 5-fold cross-validation experiments, DeepHistone (Standard) achieves higher performance than DeepHistone (DNA-only), which in turn outperforms the three baseline methods (DeepSEA, DanQ and gkm-SVM)

We first conducted 5-fold cross-validation experiments to assess the performance of our method (see Materials and methods). As shown in Table 2, for a histone marker, the auROC score averaging over the 15 epigenomes is close to 0.9, indicating the effectiveness of our method in predicting modification sites specific to a histone marker. From Fig. 2 (a), we observe that for a histone marker, the auROC score for an epigenome is typically above 0.87, though different epigenomes show fluctuations, also supporting this conclusion. Moreover, the effectiveness of our method is further supported by auPRC scores shown in Table 3 and Fig. 2 (b).

**Table 2:** Performance of different methods in terms of auROC scores

Methods	H3K4me1	H3K4me3	H3K27me3	H3K36me3	H3K9me3	H3K9ac	H3K27ac
DeepHistone (Standard)	0.9065 (0.0292)	0.9459 (0.0404)	0.8896 (0.0292)	0.8890 (0.0174)	0.8969 (0.0340)	0.9039 (0.0331)	0.9137 (0.0242)
DeepHistone (DNA-only)	0.8685 (0.0550)	0.9381 (0.0595)	0.8766 (0.0635)	0.8834 (0.0447)	0.8744 (0.0535)	0.8900 (0.1013)	0.8834 (0.0605)
DeepHistone (DNase-only)	0.8335 (0.0304)	0.8627 (0.0257)	0.7132 (0.0319)	0.7080 (0.0245)	0.7639 (0.0354)	0.8353 (0.0596)	0.8690 (0.0361)
DeepSEA	0.7828 (0.0276)	0.8987 (0.0269)	0.7829 (0.0296)	0.8167 (0.0200)	0.8341 (0.0306)	0.8330 (0.0620)	0.7934 (0.0335)
DanQ	0.7649 (0.0258)	0.8934 (0.0367)	0.7722 (0.0266)	0.8138 (0.0191)	0.8272 (0.0336)	0.8238 (0.0353)	0.7726 (0.0241)
gkm-SVM	0.6361 (0.0399)	0.8092 (0.0592)	0.6434 (0.0415)	0.6666 (0.0335)	0.7278 (0.0421)	0.7167 (0.0454)	0.6445 (0.0367)

Numbers in a cell are the mean (left) and standard deviation (right) of auROC scores over the 15 epigenomes

**Table 3:** Performance of different methods in terms of auPRC scores

Methods	H3K4me1	H3K4me3	H3K27me3	H3K36me3	H3K9me3	H3K9ac	H3K27ac
DeepHistone (Standard)	0.8116 (0.0751)	0.8432 (0.0801)	0.6655 (0.1032)	0.7551 (0.0688)	0.6779 (0.1329)	0.7271 (0.1044)	0.7714 (0.0822)
DeepHistone (DNA-only)	0.7429 (0.1268)	0.8208 (0.1348)	0.6543 (0.1382)	0.7493 (0.0754)	0.6427 (0.1424)	0.6888 (0.1835)	0.6990 (0.1535)
DeepHistone (DNase-only)	0.6664 (0.0985)	0.5962 (0.0549)	0.3219 (0.1459)	0.4043 (0.0776)	0.3489 (0.1401)	0.5701 (0.1465)	0.6673 (0.1179)
DeepSEA	0.6087 (0.0817)	0.7371 (0.0566)	0.4404 (0.1355)	0.6164 (0.0647)	0.5554 (0.1277)	0.5629 (0.1536)	0.5340 (0.1027)
DanQ	0.5805 (0.0783)	0.7303 (0.0737)	0.4233 (0.1046)	0.6178 (0.0756)	0.5420 (0.1379)	0.5502 (0.1034)	0.5015 (0.0859)
gkm-SVM	0.4237 (0.0722)	0.5858 (0.0981)	0.2774 (0.0945)	0.4069 (0.0898)	0.4221 (0.1208)	0.3889 (0.1187)	0.3340 (0.0700)

Numbers in a cell are the mean (left) and standard deviation (right) of auPRC scores over the 15 epigenomes

We then compared the performance of our method with that of the baseline approaches. Considering that our method uses both sequence and chromatin accessibility information, while the other approaches only rely on DNA sequence, we discarded the DNase module and implemented a variation of our method called DeepHistone (DNA-only). From Table 2, we observe that the mean auROC score over the 15 epigenomes for a histone marker yielded by this model, though in general has a slight drop when compared with the that generated by the original model, i.e., DeepHistone (Standard), is obviously significantly higher than all the three baseline methods (DeepSEA, DanQ, and gkm-SVM). For example, for H3K4me1, the mean auROC of the 15 epigenomes for DeepHistone (Standard) is 0.9065 ± 0.0290, while those for DeepHistone (DNA-only), DeepSEA, DanQ and gkm-SVM are 0.8685 ± 0.0550, 0.7828 ± 0.0280, 0.7649 ± 0.0260, 0.6361 ± 0.0400, respectively. This observation suggests that our method, even when using sequence information alone, is still superior over the three baseline methods in predicting modification sites specific to a histone marker.

From Fig. 2, we further confirmed this observation. Also taking H3K4me1 as an example, the median auROC of the 15 epigenomes for DeepHistone (Standard) is 0.9152 in the box plot, while those for DeepHistone (DNA-only), DeepSEA, DanQ and gkm-SVM are 0.8922, 0.8200, 0.8058, 0.6804, respectively. We then conducted a one-sided paired-sample binomial exact test to access whether the auROC scores of the 15 epigenomes yielded by a method for a histone marker is higher than those generated by another. Results show that DeepHistone (Standard) is superior to DeepHistone (DNA-only) with significant *p*-values for H3K4me1, H3K4me3, H3K9ac, and H3K27ac (all *p*-values are equal to 3.052E-05) and marginal significant *p*-values for H3K9me3 (*p*-value = 3.693E-03) and H3K27me3 (*p*-value = 5.924E-02). For H3K36me3, these two methods show no apparent difference (*p*-value = 0.500). Furthermore, DeepHistone (DNA-only) is superior to all the three baseline methods for all the 7 histone markers (all *p*-values are equal to 3.052E-05). These results further support the conclusion that our method outperforms existing baseline approaches, even when using sequence information alone.

### Contributions of the DNA and DNase modules

**Fig. 3:**
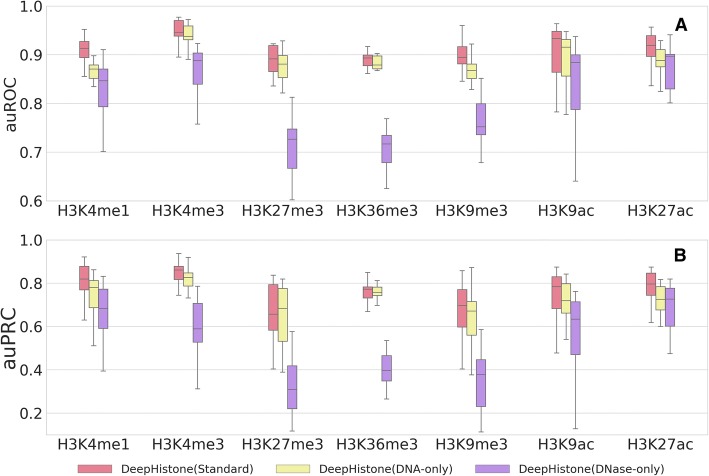
Contribution of different channels of DeepHistone. In 5-fold cross-validation experiments, DeepHistone (Standard) achieves higher performance than DeepHistone (DNA-only) and DeepHistone (DNase-only) in terms of auROC (**a**) and auPRC (**b**) scores separately, indicating that both sequence and chromatin accessibility information have a positive contribution to our method

To evaluate contributions of the sequence information and chromatin accessibility data, we discarded the DNase and DNA module from our model, yielding two variations of our method, named DeepHistone (DNA-only) and DeepHistone (DNase-only), respectively. For each of the resulting model, we repeated the 5-fold cross-validation experiments and showed the results in Tables 2 and 3, and Fig. 3.

From Table 2, we first observe that both the sequence information and chromatin accessibility data have positive contributions to the final model, because the exclusion of either information leads to a drop in the performance. For example, for H3K4me1, the discard of the chromatin accessibility information results in an average auROC of 0.8685 over the 15 epigenomes, while the exclusion of the sequence information results in an average auROC of 0.8335 over the 15 epigenomes. Both results are apparently lower than the full model, which yields an average auROC of 0.9065 over the 15 epigenomes.

We also notice that the sequence information contributes more to the final performance than the chromatin accessibility data, because the removal of the DNA module, i.e., DeepHistone (DNase-only), in general results in a larger drop in performance. To further confirmed this observation, we again conducted the aforementioned one-sided paired-sample binomial exact test to access whether the auROC scores of the 15 epigenomes yielded by DeepHistone (DNA-only) for a histone marker is higher than those generated by DeepHistone (DNase-only). Results show that the former is superior to the latter with significant *p*-values for H3K4me1, H3K4me3, H3K27me3, H3K36me3, H3K9me3 (all *p*-values are equal to 3.052E-05). For H3K9ac, the *p*-value is also significant as 4.883E-04. The only exception is H3K27ac, where the *p*-value (0.304) is not significant.

On one hand, it is not surprising that the sequence information contributes to the prediction of histone modification sites. Actually, this conclusion has been supported by abundant studies that demonstrate the effectiveness of such sequence patterns as transcription factor binding sites in the prediction of histone modification sites [14]. On the other hand, the effectiveness of chromatin accessibility information can also be explained by not only the relationship between histone methylation and DNA accessibility [31] but also the correlation between histone acetylation and chromatin status [32]. Moreover, we conjecture that chromatin accessibility information contributes less than sequence information might be due to the fact that DNase-seq data for all the 7 markers are identical for an epigenome, and signatures of chromatin accessibility may not be so strong as those of sequence.

### DeepHistone predicts histone modification sites across epigenomes

Although the above cross-validation experiments demonstrated the success of our method in the prediction of modification sites specific to a histone marker, in reality it would be more meaningful to predict histone modification sites for an epigenome that has no biological experiment conducted. We therefore proposed the following collective scoring strategy to predict status (i.e., belonging to which histone markers) of modification sites for a novel epigenome.

Given a novel epigenome and a genomic region, we would like to predict whether this region was a modification site for a histone marker, with respect to the given epigenome. To achieve this objective, we resorted to a model trained on a known epigenome to predict a probability that indicated whether this region is a modification site for the same histone marker, and we averaged all such probabilities over all known epigenomes to obtain a final prediction probability. In this procedure, the input includes the DNA sequence of the region and the chromatin accessibility data specific to the novel epigenome.

We conducted a leave-one-out experiment to evaluate the performance of our method with this strategy. Specifically, in each validation run, we selected one of the 15 epigenomes and assumed that status of modification sites in this target epigenome is unknown. Then, we applied the collective scoring strategy to recover the status of these sites by making use of the remaining 14 epigenomes. Finally, we evaluated the performance of our method in terms of the auROC and auPRC scores by using the known status of the sites in the target epigenome as the gold standard. In implementation, we took advantage of the models trained in the aforementioned 5-fold cross-validation experiments and averaged the probabilities calculated by these 5 models to obtain the prediction probability with respect to a known epigenome.

**Fig. 4:**
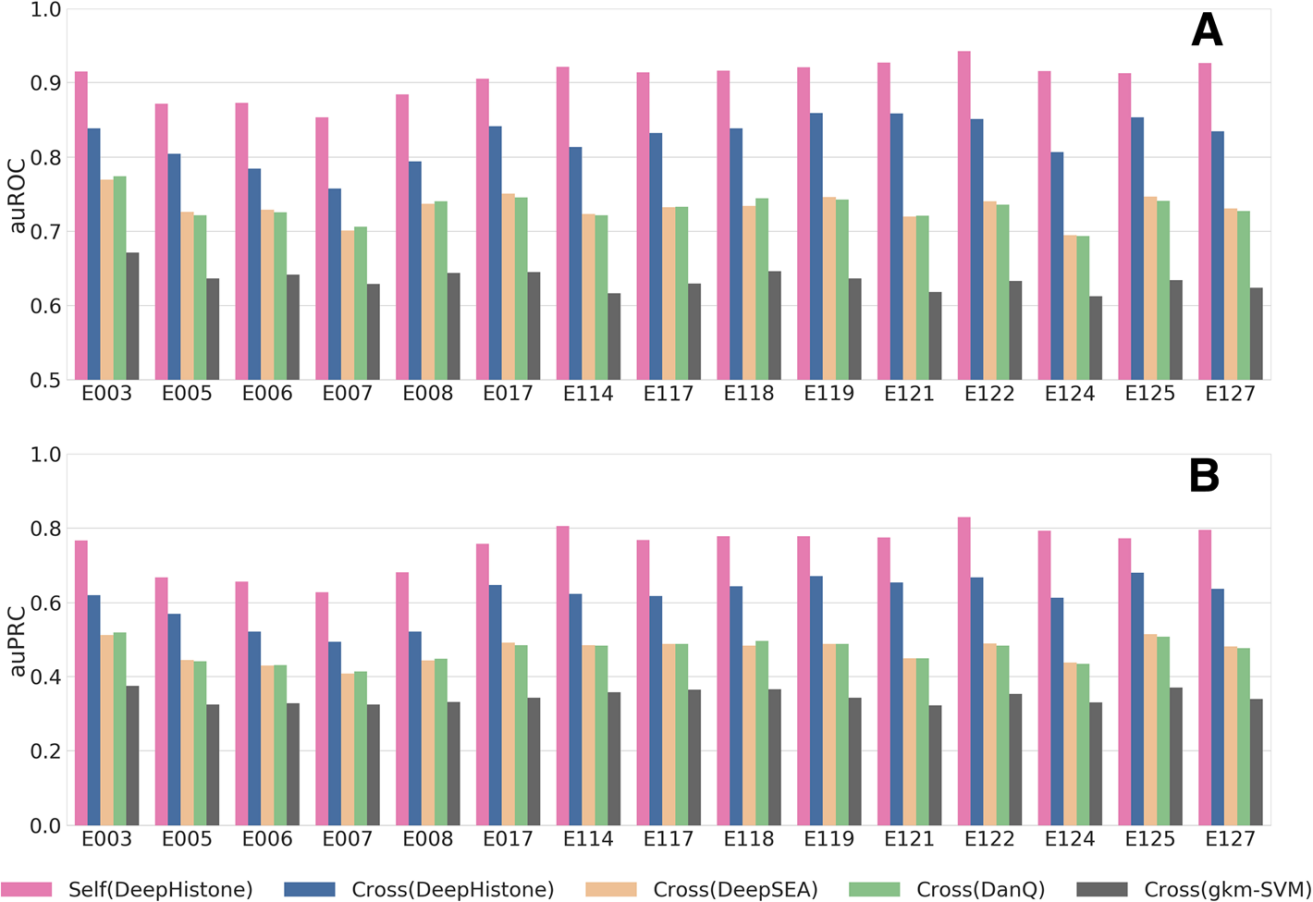
Cross cell line prediction. Each bar indicates the mean of auROC (**a**) or auPRC (**b**) of 7 histone modification markers in a certain epigenome. “Self (DeepHistone)” indicates the performance of an epigenome predicting by a DeepHistone model trained in the same epigenome. “Cross (DeepHistone)” indicates the performance of cross epigenomes prediction using DeepHistone model. Others have similar meaning

We presented the results in Fig. 4, in which both auROC and auPRC scores were averaged over the 7 histone markers to make the presentation concise. From the figure, we can clearly see that the cross-epigenome prediction by DeepHistone is effective, in that for the 15 epigenomes, the auROCs are typically above 0.8, and the auPRCs are typically above 0.6. We also notice that the cross-epigenome prediction in general exhibits lower performance than self-prediction by DeepHistone (5-fold cross-validation). This is reasonable because an epigenome may have its specific sequence codes and chromatin accessibility patterns that might not be captured by the collective scoring strategy.

When compared with the three baseline methods, DeepHistone apparently achieves higher performance for all the 15 epigenomes. For example, the average auROCs for E003 (an embryonic stem cell line) are 0.8391, 0.7697, 0.7744, and 0.6711 for DeepHistone, DeepSEA, DanQ, and gkm-SVM, respectively. Actually, DeepHistone achieves higher auROC scores than all the three baseline methods for all the 15 epigenomes. As a result, a one-sided binomial exact test against the null hypothesis that the performance of DeepHistone across the 15 epigenomes is not different from a baseline method gives significant *p*-values for all the three methods (all *p*-values are equal to 3.052E-05). This conclusion is further supported when using auPRC as the evaluation criterion.

### DeepHistone recovers TF binding motifs

**Fig. 5:**
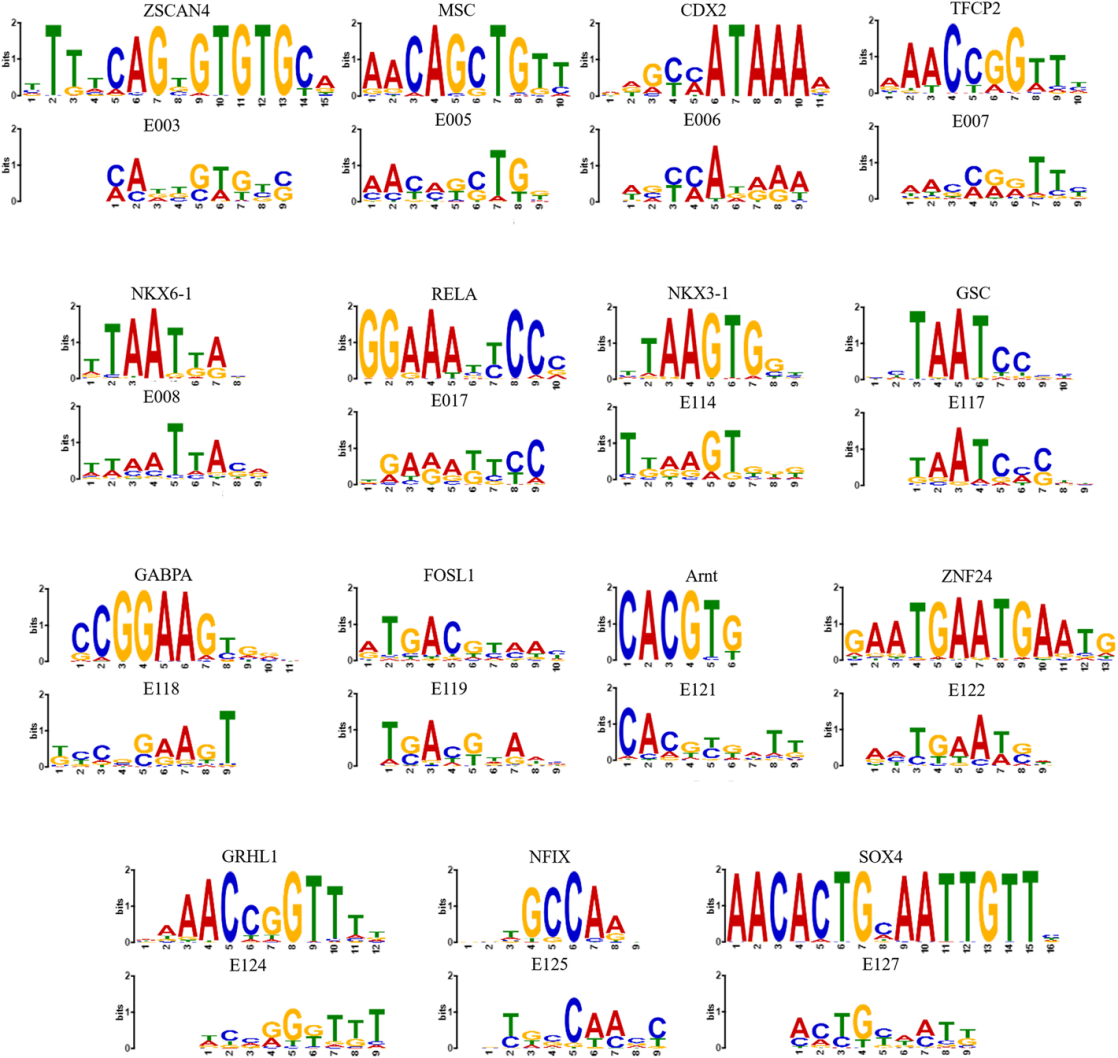
Visualization of sequence patterns learned from DeepHistone. PWMs were learned from the first convolutional layer of the DNA module. In each subgraph, the upper logo is the motif from the JASPAR database, the bottom part is the PWM learned by DeepHistone

To demonstrate sequence patterns automatically extracted by our method, we used the strategy described in Materials and methods to obtain sequence signatures (i.e., PWMs) learned from the first convolutional layer of the DNA module with respect to an epigenome. We further identified putative sequence motifs by using the tool TomTom and match these PWMs to the JASPAR database. For each epigenome, we displayed the sequence logo of one of the matched motifs in Fig. 5.

In different carcinoma cell lines, DeepHistone recovered corresponding motifs to each cell line, which showed the sensitivity of DeepHistone. In the lung carcinoma cell line (E114), DeepHistone recovered E2F3, TFAP2C and GRHL2. It has been verified that the overexpression of E2F3 transcription factor promotes the development of lung cancer [33, 34]. TFAP2C has been previously shown to promote lung tumorigenesis and aggressiveness by upregulating of TGFBR1 [35, 36]. Different from E2F3 and TFAP2C, GRHL2 can suppress tumor metastasis by regulating of transcriptional activity of RhoG in lung cancer [37]. In HeLa-S3, the cervical carcinoma cell line (E117), PROX1 and NR2F6 were found by DeepHistone. The commitment of PROX1 positive cells is an early event in cervical neoplastic progression, and the expression of PROX1 is considered as evidence of an early lymphangiogenic switch [38]. The abnormal high expression of NR2F6 in early-stage cervical cancer predicts pelvic lymph node metastasis, tumor recurrence and poor prognosis and NR2F6 might be a potential therapeutic target of cervical cancer [39]. As for hepatocellular carcinoma cell line (E118), E2F8, GABBPA and SOX11 were recovered. It has been shown that E2F8 contributes to human hepatocellular carcinoma via regulating cell proliferation [40] and is considered as a potential therapeutic target of hepatocellular cancer [41]. GABBPA inhibits metastasis of hepatocellular carcinoma [42] and SOX11 is important in the regulation of hepatocellular carcinoma cell proliferation, migration and invasion [43]. Besides, DeepHistone recovers SREBF2, HOXA5 and ZNF24 in human umbilical vein endothelial primary (HUVEC) cell line (E122) and NKX6–1 in embryonic stem cell line (E008). Those recovered transcription factors are verified to play important roles in the corresponding cell line [44–48]. To sum up, DeepHistone has the ability to recover potential functional transcription factor corresponding to specific cell line.

### DeepHistone explains functional implications of SNPs

Although genome-wide association studies (GWAS) have successfully identified thousands of single nucleotide polymorphisms (SNPs) associated with complex traits [49]**,** most of these SNPs locate outside coding regions. The explanation of the functional implications of these SNPs has thus long been a critical task in genetic studies [50]. Recently, a new technique that combines a deep and long-read ChIP-seq assay on H3K27ac with a powerful statistical test has successfully enabled the identification of histone acetylation quantitative trait loci (haQTLs) related to a lymphoblastoid epigenome. The identified SNPs exhibit highly predictive power in exploring mechanisms of autoimmune disease. We then applied DeepHistone to analyze these SNPs, demonstrating potential applications of our method.

**Fig. 6:**
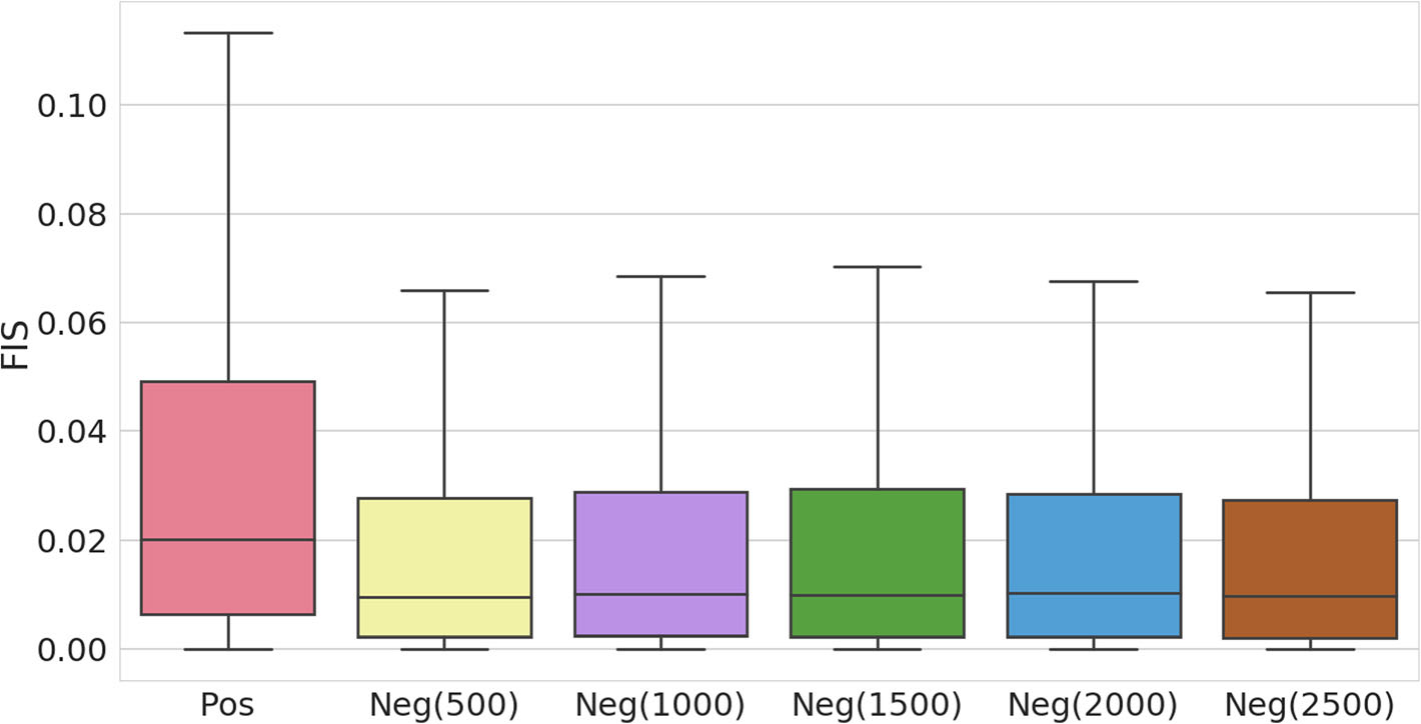
Functional implication scores (FIS) of haQTLs and their nearby SNPs. “Pos” indicates haQTLs. “Neg(500)” indicates a negative dataset containing SNPs about 500 bp away from the haQTLs. Others have similar meaning. A significant difference of functional implication scores between positive and negative datasets suggested that DeepHistone can distinguish haQTLs against their nearby SNPs

From the literature [30], we identified a positive set that includes 7497 SNPs (haQTLs) specific to H3K27ac in the lymphoblastoid epigenome (E116) and appearing in the 1000 genomes project [51]. Meanwhile, we generated a negative control set that includes the same number of SNPs as the positive one by identifying for each haQTL a SNP that locates about 500 bp away, also from the 1000 genomes project. We then used the formulation detailed in Materials and methods to calculate functional implication scores for the identified SNPs and compared whether scores for positive SNPs are significantly different from those for negative ones. The results, as shown in Fig. 6, clearly show that the haQTLs tend to have higher functional implication scores than the control SNPs. A one-sided Wilcoxon rank sum test against the null hypothesis that the median score of these two sets of SNPs are identical yield a very significant *p*-value of 1.369E-140, strongly support the conclusion that haQTLs have higher functional implication scores. In other words, these SNPs are more likely to change the function of the lymphoblastoid epigenome, and thus are more likely to be responsible to a phenotype. We further generated other four control sets in which a SNP is required to be 1000, 1500, 2000 and 2500 bp away from a haQTL. The results, as shown in Fig. 6, give us a similar conclusion. All these results suggest that our method has the potential ability to discriminant SNPs responsible for a certain phenotype from their nearby genetic variants.

## Conclusions and discussion

We have proposed a deep learning framework named DeepHistone to integrate DNA sequence information and chromatin accessibility data for predicting histone modification sites. Through comprehensive validation experiments regarding 7 histone markers and 15 epigenomes, we have shown that our approach is superior to several baseline methods in discriminating among modification sites specific to different histone markers, capable of making predictions across epigenomes, interpretable in extracted sequence features, and applicable to the explanation of functional implications of genetic variants.

The success of our method can be attributed to the combination of the following facts. First, we have designed a novel deep neural network model with the incorporation of state-of-the-art techniques in the deep learning community. Particularly, the densely connected architecture effectively overcomes such problems as the vanishing gradient and overfitting, and greatly improves the prediction accuracy. Second, besides sequence information, we have also incorporated chromatin accessibility data into our model. These two types of information can then complement each other in our neural network model to capture subtle signals towards the accurate prediction of histone modification sites.

Certainly, our work can be further improved in several aspects. First, resorting to an embedding representation of DNA sequences instead of using the one-hot encoding may further improve the prediction accuracy [21]. Second, considering the sequential natural a DNA fragments, the incorporation of a recurrent neural network architecture, especially long short-term memory units, may further improve the performance of our method [18, 21]. Third, instead of scanning sequence motifs from the beginning using convolutional kernels, it is also possible to incorporate sequence patterns and design a hybrid network architecture [20]. Fourth, we used DNase-seq peaks from Roadmap to quantify chromatin accessibility. This treatment, though simple, may not be precise. Fifty, besides chromatin accessibility data, it is also worth to consider the integration of plenty of gene expression data. Finally, besides our current formulation of predicting for a certain epigenome putative modification sites specific to different histone markers, it will also be beneficial to formulate the problem from the perspective of predicting for a fixed histone marker putative sites for different epigenomes.

## Abbreviations

auPRC: Area under the Precision-Recall curve
auROC: Area under the Receiver Operating Characteristic curve
CHIP-seq: Chromatin immunoprecipitation followed by sequencing
DNase-seq: DNase I hypersensitive sites sequencing
GWAS: Genome wide association study
haQTL: Histone acetylation quantitative trait loci
SNP: Single nucleoid polymorphism
TF: Transcription factor

## References

[1] Allfrey VG, Faulkner R, Mirsky AE (1964). Acetylation and methylation of histones and their possible role in the regulation of RNA synthesis. Proc Natl Acad Sci U S A.

[2] Luger K, Mäder AW, Richmond RK, Sargent DF, Richmond TJ (1997). Crystal structure of the nucleosome core particle at 2.8 Å resolution. Nature.

[3] Peterson CL, Laniel MA (2004). Histones and histone modifications. Curr Biol.

[4] Bannister AJ, Kouzarides T (2011). Regulation of chromatin by histone modifications. Cell Res.

[5] Brehove M, Wang T, North J, Luo Y, Dreher SJ, Shimko JC, Ottesen JJ, Luger K, Poirier MG (2015). Histone core phosphorylation regulates DNA accessibility. J Biol Chem.

[6] Cheung P, Allis CD, Sassone-Corsi P (2000). Signaling to chromatin through histone modifications. Cell.

[7] Binder H, Steiner L, Przybilla J, Rohlf T, Prohaska S, Galle J (2013). Transcriptional regulation by histone modifications: towards a theory of chromatin re-organization during stem cell differentiation. Phys Biol.

[8] Kouzarides T (2007). Chromatin modifications and their function. Cell.

[9] Kristeleit R, Stimson L, Workman P, Aherne W (2004). Histone modification enzymes: novel targets for cancer drugs. Expert Opin Emerg Drugs.

[10] O’Geen H, Echipare L, Farnham PJ (2011). Using ChIP-Seq technology to generate high-resolution profiles of histone modifications. Methods Mol Biol.

[11] Barski A, Cuddapah S, Cui K, Roh T-Y, Schones DE, Wang Z, Wei G, Chepelev I, Zhao K (2007). High-resolution profiling of histone methylations in the human genome. Cell.

[12] Consortium EP (2004). The ENCODE (ENCyclopedia of DNA elements) project. Science.

[13] Roadmap Epigenomics C, Kundaje A, Meuleman W, Ernst J, Bilenky M, Yen A, Heravi-Moussavi A, Kheradpour P, Zhang Z, Wang J (2015). Integrative analysis of 111 reference human epigenomes. Nature.

[14] Benveniste D, Sonntag H-J, Sanguinetti G, Sproul D (2014). Transcription factor binding predicts histone modifications in human cell lines. Proc Natl Acad Sci U S A.

[15] Karlic R, Chung HR, Lasserre J, Vlahovicek K, Vingron M (2010). Histone modification levels are predictive for gene expression. Proc Natl Acad Sci U S A.

[16] Alipanahi B, Delong A, Weirauch MT, Frey BJ (2015). Predicting the sequence specificities of DNA- and RNA-binding proteins by deep learning. Nat Biotechnol.

[17] Zhou J, Troyanskaya OG (2015). Predicting effects of noncoding variants with deep learning-based sequence model. Nat Methods.

[18] Quang D, Xie X (2016). DanQ: a hybrid convolutional and recurrent deep neural network for quantifying the function of DNA sequences. Nucleic Acids Res.

[19] Min X, Zeng W, Chen S, Chen N, Chen T, Jiang R (2017). Predicting enhancers with deep convolutional neural networks. BMC Bioinformatics.

[20] Liu Q, Xia F, Yin Q, Jiang R (2018). Chromatin accessibility prediction via a hybrid deep convolutional neural network. Bioinformatics.

[21] Min X, Zeng W, Chen N, Chen T, Jiang R (2017). Chromatin accessibility prediction via convolutional long short-term memory networks with k-mer embedding. Bioinformatics.

[22] Li W, Wong WH, Jiang R: DeepTACT: predicting high-resolution chromatin contacts via bootstrapping deep learning. bioRxiv 2018. 10.1093/nar/gkz167 PMC6547469 30869141

[23] Brykczynska U, Hisano M, Erkek S, Ramos L, Oakeley EJ, Roloff TC, Beisel C, Schubeler D, Stadler MB, Peters AH (2010). Repressive and active histone methylation mark distinct promoters in human and mouse spermatozoa. Nat Struct Mol Biol.

[24] Huang G, Liu Z, Weinberger KQ: Densely connected convolutional networks. 2017 IEEE Conference on Computer Vision and Pattern Recognition (CVPR) 2017:2261–2269.

[25] Paszke A, Gross S, Chintala S, Chanan G, Yang E, DeVito Z, Lin Z, Desmaison A, Antiga L, Lerer A (2017). Automatic differentiation in pytorch.

[26] Kingma DP, Ba J: Adam: A method for stochastic optimization. CoRR 2014, abs/1412.6980.

[27] Lee D, Gorkin DU, Baker M, Strober BJ, Asoni AL, Mccallion AS, Beer MA (2015). A method to predict the impact of regulatory variants from DNA sequence. Nat Genet.

[28] Khan A, Fornes O, Stigliani A, Gheorghe M, Castro-Mondragon JA, van der Lee R, Bessy A, Cheneby J, Kulkarni SR, Tan G et al: JASPAR 2018: update of the open-access database of transcription factor binding profiles and its web framework. Nucleic Acids Res 2018, 46(D1):D260-D266. 10.1093/nar/gkx1126 PMC5753243 29140473

[29] Gupta S, Stamatoyannopoulos JA, Bailey TL, Noble WS (2007). Quantifying similarity between motifs. Genome Biol.

[30] del Rosario RC, Poschmann J, Rouam SL, Png E, Khor CC, Hibberd ML, Prabhakar S (2015). Sensitive detection of chromatin-altering polymorphisms reveals autoimmune disease mechanisms. Nat Methods.

[31] Cui P, Li J, Sun B, Zhang M, Lian B, Li Y, Xie L (2013). A quantitative analysis of the impact on chromatin accessibility by histone modifications and binding of transcription factors in DNase I hypersensitive sites. Biomed Res Int.

[32] Görisch SM, Wachsmuth M, Tóth KF, Lichter P, Rippe K (2005). Histone acetylation increases chromatin accessibility. J Cell Sci.

[33] Cooper CS, Nicholson AG, Foster C, Dodson A, Edwards S, Fletcher A, Roe T, Clark J, Joshi A, Norman A (2006). Nuclear overexpression of the E2F3 transcription factor in human lung cancer. Lung Cancer.

[34] Trikha P, Sharma N, Pena C, Reyes A, Pécot T, Khurshid S, Rawahneh M, Moffitt J, Stephens JA, Fernandez SA (2015). E2f3 in tumor macrophages promotes lung metastasis. Oncogene.

[35] Kang J, Kim W, Lee S, Kwon D, Chun J, Son B, Kim E, Lee J, Youn H, Youn B (2017). TFAP2C promotes lung tumorigenesis and aggressiveness through miR-183-and miR-33a-mediated cell cycle regulation. Oncogene.

[36] Kim W, Kim E, Lee S, Kim D, Chun J, Park KH, Youn H, Youn B (2016). TFAP2C-mediated upregulation of TGFBR1 promotes lung tumorigenesis and epithelial–mesenchymal transition. Exp Mol Med.

[37] Pan X, Zhang R, Xie C, Gan M, Yao S, Yao Y, Jin J, Han T, Huang Y, Gong Y (2017). GRHL2 suppresses tumor metastasis via regulation of transcriptional activity of RhoG in non-small cell lung cancer. Am J Transl Res.

[38] Cimpean AM, Mazuru V, Saptefrati L, Ceausu R, Raica M (2012). Prox 1, VEGF-C and VEGFR3 expression during cervical neoplasia progression as evidence of an early lymphangiogenic switch. Histol Histopathol.

[39] Niu C, Sun X, Zhang W, Li H, Xu L, Li J, Xu B, Zhang Y. NR2F6 expression correlates with pelvic lymph node metastasis and poor prognosis in early-stage cervical Cancer. Int J Mol Sci. 2016;17(10):1694. 10.3390/ijms17101694 PMC5085726 27775588

[40] Deng Q, Wang Q, Zong WY, Zheng DL, Wen YX, Wang KS, Teng XM, Zhang X, Huang J, Han ZG (2010). E2F8 contributes to human hepatocellular carcinoma via regulating cell proliferation. Cancer Res.

[41] Lv Y, Xiao J, Liu J, Xing F (2017). E2F8 is a potential therapeutic target for hepatocellular carcinoma. J Cancer.

[42] Zhang S, Zhang K, Ji P, Zheng X, Jin J, Feng M, Liu P (2017). GABPA predicts prognosis and inhibits metastasis of hepatocellular carcinoma. BMC Cancer.

[43] Wang Z, Li Z, Zhu J (2017). Negative regulation of SOX11 in hepatocellular carcinoma. Int J Clin Exp Med.

[44] Xiao H, Lu M, Lin TY, Chen Z, Chen G, Wang WC, Marin T, Shentu TP, Wen L, Gongol B (2013). Sterol regulatory element binding protein 2 activation of NLRP3 inflammasome in endothelium mediates hemodynamic-induced atherosclerosis susceptibility. Circulation.

[45] Zeng L, Liao H, Liu Y, Lee TS, Zhu M, Wang X, Stemerman MB, Zhu Y, Shyy JY. Sterol-responsive element-binding protein (SREBP) 2 down-regulates ATP-binding cassette transporter A1 in vascular endothelial cells: a novel role of SREBP in regulating cholesterol metabolism. J Biol Chem. 2004;279(47):48801-7. 10.1074/jbc.M407817200 15358760

[46] Fork C, Gu L, Hitzel J, Josipovic I, Hu J, SzeKa Wong M, Ponomareva Y, Albert M, Schmitz SU, Uchida S (2015). Epigenetic regulation of angiogenesis by JARID1B-induced repression of HOXA5. Arterioscler Thromb Vasc Biol.

[47] Jia D, Huang L, Bischoff J, Moses MA (2015). The endogenous zinc finger transcription factor, ZNF24, modulates the angiogenic potential of human microvascular endothelial cells. FASEB J.

[48] Rezania A, Bruin JE, Xu J, Narayan K, Fox JK, O'Neil JJ, Kieffer TJ (2013). Enrichment of human embryonic stem cell-derived NKX6.1-expressing pancreatic progenitor cells accelerates the maturation of insulin-secreting cells in vivo. Stem Cells.

[49] MacArthur J, Bowler E, Cerezo M, Gil L, Hall P, Hastings E, Junkins H, McMahon A, Milano A, Morales J (2016). The new NHGRI-EBI catalog of published genome-wide association studies (GWAS catalog). Nucleic Acids Res.

[50] Wu M, Lin Z, Ma S, Chen T, Jiang R, Wong WH (2017). Simultaneous inference of phenotype-associated genes and relevant tissues from GWAS data via Bayesian integration of multiple tissue-specific gene networks. J Mol Cell Biol.

[51] Genomes Project C, Auton A, Brooks LD, Durbin RM, Garrison EP, Kang HM, Korbel JO, Marchini JL, McCarthy S, McVean GA (2015). A global reference for human genetic variation. Nature.

